# Mental symptoms in post-COVID syndrome and post-COVID-19 vaccination syndrome: results of a representative population survey

**DOI:** 10.3389/fpubh.2025.1623757

**Published:** 2025-08-04

**Authors:** Andreas Czaplicki, Leonie Hipper, Ulrich Hegerl

**Affiliations:** ^1^German Foundation for Depression and Suicide Prevention, Leipzig, Germany; ^2^Department of Psychiatry, Psychosomatic Medicine and Psychotherapy, University Hospital Frankfurt, Goethe University Frankfurt (Goethe Research Professorship), Frankfurt am Main, Germany

**Keywords:** post-COVID, COVID-vaccine, COVID 19, differential diagnosis, mental health, depression, mental symptoms, population survey

## Abstract

**Background:**

Mental symptoms such as fatigue, concentration difficulties, and sleep disorders are frequently reported in both Post-COVID Syndrome (PCS) and Post-COVID-19 Vaccination Syndrome (PCVS). Although symptom profiles may overlap, comparative epidemiological data from the general population are limited.

**Methods:**

We conducted a representative online survey of 4,632 adults in Germany and investigated mental symptoms associated with PCS and PCVS, including fatigue, cognitive problems, sleep disturbances, impaired performance and depressed mood. Socio-demographic factors, vaccination status, vaccine type and number of vaccinations were analysed.

**Results:**

The prevalence of self-reported mental symptoms of PCS was 12.1% among respondents, with women and younger individuals more commonly affected. The prevalence of self-reported PCVS was 12.6% among vaccinated individuals, with no significant gender differences, but fewer cognitive symptoms in older adults. Respondents with only one vaccination reported the highest rate of PCVS symptoms (20.8%), which decreased to 8.9% for those with four or more vaccinations. Differences between vaccine types were small overall, but non-mRNA vaccines were associated with a slightly higher rate of sleep and concentration problems.

**Conclusion:**

Mental symptoms are relatively common after both SARS-CoV-2 infection and vaccination and show overlap, especially fatigue. These findings highlight how difficult it is to distinguish PCS from PCVS and emphasize the need to consider alternative explanations, such as undiagnosed depressive disorders, in clinical assessments.

## 1 Introduction

Mental symptoms such as fatigue, cognitive impairment and sleep disturbances are frequently observed in both post-COVID syndrome (PCS) and post-COVID-19 vaccination syndrome (PCVS) (1). PCS is defined as a condition characterised by unexpected symptoms that persist for at least 3 months after an acute COVID-19 infection and continue for at least another 3 months (2). Population-based studies report different prevalence rates of PCS, ranging from 7.3 to 28.5% (3–6). In a systematic review, insomnia, fatigue and anxiety were identified as the most common PCS-related symptoms, with pooled prevalence rates of 27.4, 24.4 and 19.1%, respectively, (7). Other symptoms include memory problems, ‘brain fog’, depressed mood and anxiety (8–10).

In contrast, PCVS refers to adverse mental or physical reactions following COVID-19 vaccination. Although the term “post-vac” is widely used in public discourse to describe long-lasting symptoms after vaccination, it lacks formal medical definition or diagnostic criteria (11). Official data suggest a low rate of documented adverse effects: the European Medicines Agency (EMA) reports a 0.2% rate of side effects (12), and the Paul Ehrlich Institute (PEI) reports a 0.52% rate of suspected adverse reactions following COVID-19 vaccination (11). However, these data are likely subject to underreporting, given the passive nature of current surveillance systems and the high threshold for physician-initiated reporting.

While the somatic side effects of COVID-19 vaccines have been extensively studied (13–17), data on mental symptoms following vaccination remain limited. Existing studies often focus on fatigue as a systemic symptom (11, 18) or are restricted to specific occupational groups such as healthcare professionals (19–21). To date, there is a lack of population-based data on mental symptoms after COVID-19 vaccination, particularly in relation to vaccination dose count and vaccine type (mRNA vs. non-mRNA). Preliminary national data from Germany suggest a slightly higher rate of chronic fatigue following non-mRNA vaccines, although these figures are difficult to interpret given the low proportion of non-mRNA vaccines administered (11, 22).

Given the potential for both underreporting and overreporting in spontaneous reporting systems, representative population-based surveys offer a valuable complement to official surveillance data. They can help clarify the epidemiological scope and symptom profiles of both PCS and PCVS, and improve understanding of their possible overlap.

This representative survey of the adult German population analysed how common mental symptoms are in PCS and PCVS and whether there are subgroups with an enhanced risk in each case. Differences in the epidemiology and symptomatology of PCVS with regard to the number of vaccinations and the type of vaccination (mRNA vs. non-mRNA) were investigated.

## 2 Materials and methods

### 2.1 Study design

The presented results are based on an epidemiological, non-interventional study with n = 4,628 participants. The survey period was from August 28 to September 8, 2023.

### 2.2 Study population and sample characteristics

Study population is the German resident population aged 18 to 69 years. Persons under the age of 18 and persons over the age of 60 were not surveyed. Respondents had to be resident in Germany. There were no other inclusion or exclusion criteria for the respondents.

A multiple stratified quota sample with 70 quota cells using the interleaved characteristics of gender, age and state/province groups was assessed. The basis for the specifications in the quota cells were the current population updates of the Federal Statistical Office.

The sample size was not calculated *a priori*. However, a basis of approximately 5,000 interviews had proven to be a good basis for analysing subgroups within the population in previous surveys with regard to the probability of error, confidence interval and standard error.

Of the people contacted, 15.6% responded to the invitation letter and called up the questionnaire (gross sample); the remaining 84.4% did not respond. The gross sample was reduced for various reasons: Participants were rejected because the corresponding quota cells were already filled with a sufficient number of respondents; because the interview was cancelled; because interviews were sorted out after quality control. The response rate was therefore 12.2%.

### 2.3 Data collection

The survey was conducted as an online survey with a structured questionnaire on our behalf by the survey institute Bilendi. Bilendi has a pool of people who have declared their general willingness to participate in online surveys. Basic data is available from these people that corresponds to the characteristics of our quota cells. From this pool of respondents, participants were successively selected at random within the specified quota cells and invited to take part in the survey.

The gender, age and state/province groups relevant to the quota were queried at the beginning of the interview. If the target for the respective quota cell had not yet been met, the interview was started. If the specified number of interviews had already been reached in a quota cell, the interviewees received a friendly cancellation and the interview was ended prematurely. The topic of the survey was not specified in order to prevent a participant bias.

### 2.4 Measures

*Self-reported PCS*: Respondents were asked whether, in their own opinion, they had suffered from PCS using the following wording: “In your opinion, have you suffered from long COVID? Long COVID refers to symptoms that persist, worsen or reappear more than four weeks after infection with the coronavirus, for which there is no other explanation. Long-COVID also includes post-COVID syndrome, which refers to symptoms that appear or persist three months or longer after a coronavirus infection.” The possible answers were ‘Yes’, ‘No’ or ‘Do not know’. The following complaints were considered to be mental symptoms: restricted performance, tiredness/fatigue/weakness, concentration and memory problems, sleep disorders or a depressed mood/depression.

*Self-reported PCVS*: Respondents who were vaccinated against COVID were asked about PCVS with the following question: “In your opinion, have you experienced any vaccination side effects (lasting consequences) from a COVID vaccination?” The possible answers here were also ‘Yes’, ‘No’ or ‘Do not know’.

The symptoms of PCVS were queried as follows: “What symptoms do you suffer from? (multiple answers possible): shortness of breath, tiredness/fatigue/weakness, concentration and memory problems, reduced performance, muscle pain, cough/coughing irritation, sleep disorders, palpitations/heart palpitations, loss/change of taste and/or smell, headache, chest pain, tightness in the chest, joint pain, depressed mood/depression, other complaints.” These symptoms were described as symptoms of Long COVID by > = 50% of respondents in a Delphi study. Participants in the Delphi study included clinical researchers, patients, members of the WHO Research Working Group for the Clinical Characterization and Management of COVID-19, members of the WHO Clinical Network for COVID-19, members of a Long COVID SOS patient group, and clinicians and patients nominated by WHO officials (23).

*Number of vaccinations*: The number of COVID-19 vaccinations received was queried as follows: “Have you been vaccinated against COVID?” The possible answers were: ‘yes, once’, ‘twice’, ‘three times’, ‘four times’, ‘five times’, ‘more than five times’, ‘no’ and ‘do not know’.

*Type of vaccine*: The following question was used to determine which vaccine was used for the vaccinations: “Which vaccine(s) were you vaccinated with? (multiple answers possible).” Possible answers were the ‘Comirnaty from BioNTech/Pfizer (mRNA vaccine)’, ‘Spikevax from Moderna (mRNA vaccine)’, ‘Jcovden from Janssen- Cilag’, ‘Vaxzevria from AstraZeneca’, ‘VidPrevtyn from Sanofi’, ‘Nuvaxovid from Novavax’, ‘COVID-19 Vaccine Valneva from Valneva’, ‘Bimervax from HIPRA’, ‘Sputnik’ and ‘other and namely (open information)’.

The questions were developed internally at our research center. The instruments were not validated. They are mainly descriptive questions and questions on the respondents’ self-assessment.

### 2.5 Statistical analysis

The self-reported PCS was analysed for the subgroups of gender and age group. The self-reported PCVS was analysed for the subgroups of gender, age group, vaccine and number of vaccinations.

Univariable logistic regression models were used to analyse the relationships between socio-demographic variables and the outcome of interest. Odds ratios (ORs) with corresponding 95% confidence intervals (CIs) were calculated using appropriate reference categories. In addition to the regression results, weighted prevalence estimates and their 95% CIs were provided to account for the sample design. Survey weights were used to adjust for possible differences between the sample and the underlying population structure. All statistical tests were two-sided; a *p*-value <0.05 was considered statistically significant. Statistical tests were corrected for multiple testing using the Bonferroni method. Data analysis was performed using IBM SPSS Statistics, version 29.0.2.0 (IBM Corp., Armonk, NY, United States).

### 2.6 Ethical approval

The study protocol has been reviewed by the Ethics Committee of the Department of Medicine at Goethe University Frankfurt (file number 2023–1174). In a certificate dated 17^th^ February 2023, it was confirmed that there was no obligation to obtain ethics approval for the anonymous data collection.

## 3 Results

### 3.1 Demographic characteristics

The total sample comprised 4,628 individuals. Gender distribution was nearly balanced, with 2,299 females (49.7%) and 2,329 males (50.3%). Specifically, 913 individuals (19.7%) were aged 18–29 years, 908 (19.6%) were 30–39 years, 835 (18.0%) were 40–49 years, 1,048 (22.6%) were 50–59 years, and 924 (20.0%) were 60–69 years.

### 3.2 Epidemiology of mental symptoms of PCS

**Figure 1:**
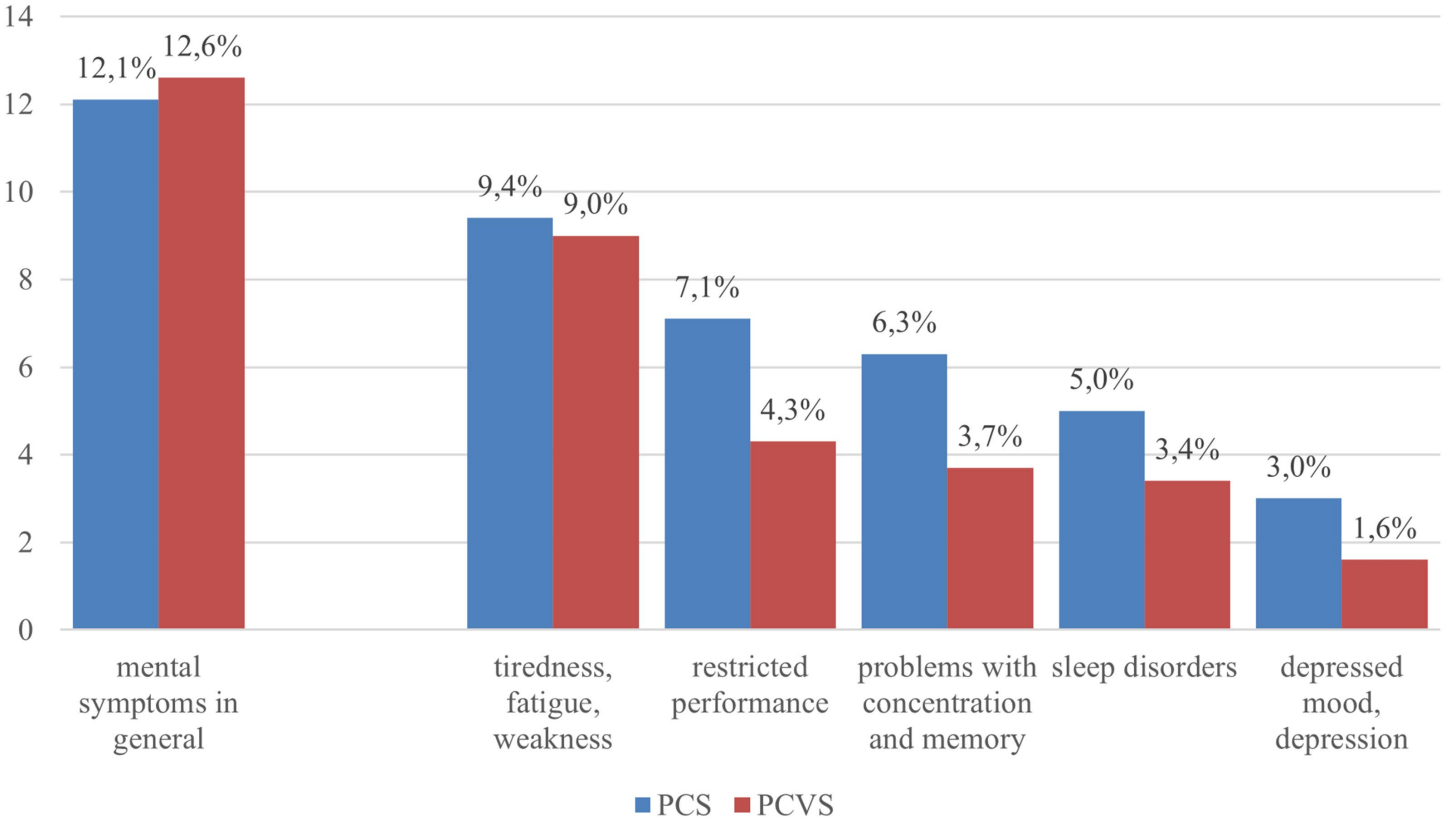
Reported mental symptoms in both the context of PCS and PCVS (summary and for 5 symptoms separately). Blue bar: all respondents (*N* = 4,628); red bar: all persons with vaccination (*N* = 4,049).

In the survey, 12.1% (*n* = 560) of all respondents mentioned at least one of the mental symptoms of PCS that we presented in the survey. The prevalence of different self-reported symptoms are shown in Figure 1.

Females reported mental symptoms of PCS more often (13.5%; *n* = 311) compared to males (10.7%; *n* = 249, *p* = 0.0032). People aged 18 to 29 years were the most likely to report psychological symptoms of PCS (14.0%, *n* = 128), while people aged 60 to 69 years were the least likely to report psychological symptoms of PCS (9.4%, *n* = 87). In relation to the gender of the respondents, this result is also evident in the five different symptoms that the respondents were asked to choose from. All symptoms were reported significantly more frequently by women than by men. Sleep disorders considered to be due to PCS were significant but also slightly higher in the 50–59 years age group (6.2%, *p* = 0.0269; Supplementary Tables S1, S2).

### 3.3 Epidemiology of mental vaccination side effects

In the survey, 88.1% (*n* = 4,079; 87.9% males, 88.4% females) of respondents stated that they had been vaccinated against SARS-CoV2.

Of the respondents, 12.6% (*n* = 512) reported mental side effects of the COVID-19 vaccination.

Mental problems as side effects of COVID vaccination were mentioned for the individual symptoms: tiredness/fatigue/weakness (9.0%), restricted performance (4.3%), concentration and memory problems (3.7%), sleep disorders (3.4%) or a depressed mood/depression (1.5%).

Regarding mental PCVS, no gender differences were found (Supplementary Table S3). Only the tiredness/fatigue/weakness symptoms were reported less frequently by men (7.8%) than by women (10.2%, *p* = 0.0069). Age-related differences were only found for the problems with concentration and memory; here, older groups reported corresponding side effects less frequently (Supplementary Tables S4–S8).

### 3.4 Effects of the number of vaccinations on mental symptoms

Of those vaccinated, 2.6% (*n* = 106) reported one vaccination, 21.2% (*n* = 865) reported two vaccinations, 59.2% (*n* = 2,415) reported three vaccinations, and 17.0% (*n* = 693) reported four or more vaccinations. The reported mental side effects of the COVID vaccination decreased with the number of vaccinations: 20.8% (*n* = 22) with one vaccination, decreasing to 8.9% (n = 62) with four or more vaccinations.

The number of COVID vaccinations showed significant effects for almost all of the mental symptoms surveyed. Respondents with either one or two vaccinations reported these symptoms more frequently compared to respondents having had four or more vaccinations (Supplementary Tables S3–S7)

### 3.5 Differences in reported side effects between mRNA and non-mRNA vaccines

Overall, 92.2% (*n* = 3,762) of all vaccinated persons were vaccinated at least once with an mRNA vaccine. Of those vaccinated, 81.6% received a vaccination with the “Comirnaty” vaccine from BioNTech/Pfizer, 32.1% reported at least one vaccination with “Spikevax” from Moderna, 17.7% with “Vaxzevria” from AstraZeneca, and 4.9% with the vaccine “Jcovden” from Janssen-Cilag. All other vaccines were administered only rarely. A few respondents also reported vaccinations with unauthorized vaccines or vaccines with other indications, e.g., Bionical, Asenica, Sinovax.

We distinguish between three types of vaccines: people who received only mRNA vaccine, people who received only non-mRNA vaccine and people who received both vaccines. Types of vaccine (mRNA only, non-mRNA only, both) showed no significant differences when all mental symptoms surveyed were taken together. However, differences could be seen when differentiating between the various mental symptoms. Recipients of non-mRNA vaccines reported tiredness/fatigue/weakness more frequently (12.7%) than others; the same applied to problems with concentration and memory (9.2%) and sleep disorders (7.4%).

## 4 Discussion

*Self-reported Mental Symptoms Following COVID-19 Infection (PCS)*: In our study, 12.1% of the general population reported experiencing at least one mental symptom consistent with Post-COVID Syndrome (PCS). This prevalence is lower than that reported in clinical studies, likely because our data reflect a general population sample, while clinical data often overrepresent symptomatic individuals seeking care. In both our and previous studies, fatigue emerged as the most frequently reported symptom (7–10).

Our results confirmed a higher prevalence of mental PCS symptoms among women compared to men, which aligns with prior research showing that women are disproportionately affected by PCS (8, 24). The reasons for this gender difference are likely multifactorial, potentially involving biological, hormonal, and psychosocial components. This is consistent with findings that women are at higher risk of Long Covid and psychiatric disorders such as anxiety and depression (25).

In terms of age, younger respondents were more frequently affected by mental PCS symptoms than older individuals, an inverse age trend that warrants further investigation into possible resilience factors among older adults or differing symptom attribution.

*Self-reported Mental Symptoms Following COVID-19 Vaccination (PCVS)*: Mental symptoms attributed to Post-COVID-19 Vaccination Syndrome (PCVS) were reported by 12.6% of vaccinated individuals in the study. Similar to PCS, fatigue was the most frequently mentioned symptom, although the overall symptom pattern differed somewhat. In contrast to PCS, no significant gender differences were observed for PCVS, with the exception of fatigue, which was reported more often by women than men. Minor age-related effects were observed for concentration and memory problems, which were reported less frequently among older respondents. This outcome is consistent with the results of a population-based cohort study in South Korea, which found a higher incidence of psychiatric consequences of COVID-19 vaccination in women whereas older people showed a lower rate in symptoms of depression and anxiety disorders after COVID-19 vaccination (26).

The self-reported prevalence of PCVS symptoms here is notably higher than the 0.52% rate of adverse events documented by the Paul-Ehrlich-Institute (PEI) [1111]. Several factors may explain this discrepancy, including underreporting by the general public and healthcare providers, lack of awareness of reporting systems, and the complexity of the reporting process. Our findings suggest that official data likely underestimate the real burden of mental side effects related to COVID-19 vaccination.

*Number of Vaccinations and Mental Side Effects*: A novel and particularly relevant finding of this study is the inverse relationship between the number of COVID-19 vaccinations received and the prevalence of reported mental PCVS symptoms. Individuals with only one vaccination reported the highest rate of mental side effects (20.8%), whereas those with four or more vaccinations reported significantly fewer mental symptoms (8.9%). This result contrasts with the results of a cohort study by Shrestha et al., which showed that booster vaccinations increased the incidence of PCVS symptoms (27). The pattern found in our study could be partially explained by self-selection, where individuals experiencing side effects after early vaccinations may have been less likely to continue with further doses. Emerging literature also highlights a strong influence of psychological mechanisms in post-vaccine reactions. Nocebo effects have been observed, especially in individuals with pre-existing anxiety or mood disorders (28).

*Vaccine Type and Mental Symptom Patterns*: Although no significant differences in overall prevalence of mental PCVS symptoms were observed between recipients of mRNA and non-mRNA vaccines (12.5% vs. 12.9%), specific symptom patterns differed. Concentration and memory problems, impaired performance, and sleep disorders were more frequently reported by those who received non-mRNA vaccines. This could reflect differences in vaccine-induced immune responses (28).

In contrast to official data suggesting slightly more fatigue after non-mRNA vaccines, our study did not find significant differences in fatigue or depressive symptoms between vaccine types. (11, 29). To our knowledge, comparative symptom profiles by vaccine type have not been previously documented and merit further study.

*Diagnostic Challenges and Broader Interpretations*: One of the key implications of our findings is the difficulty of clinically distinguishing PCS from PCVS, particularly given the overlap in symptomatology and timing. This implication is also emphasized by Shrestha et al. (29) who were able to show that the symptom patterns of PCVS and PCS are considerably similar. Fatigue, for example, may be linked to persistent immune activation following infection or vaccination, or could reflect other pathophysiological processes such as myocarditis.

Compounding this issue is the fact that many of the reported mental symptoms—such as fatigue, sleep problems, and cognitive impairment—are also core features of common mental disorders, especially depression.

The possibility that symptoms attributed to PCS or PCVS may, in some cases, reflect undiagnosed depressive disorders deserves particular attention. Previous studies have shown that individuals with depression frequently misattribute their symptoms to physical causes, and the context of COVID-19 and vaccination offers ready explanations for such misattributions. The higher reporting of symptoms by women is consistent with the known higher prevalence of depression in females (30, 31).

*Conclusion of Discussion*: Overall, our findings indicate that mental symptoms attributed to both PCS and PCVS are relatively common in the general population. The overlap in symptom profiles, combined with diagnostic ambiguity and possible underreporting in official systems, underscores the importance of cautious interpretation in both clinical and public health contexts. Further research is needed to disentangle PCS, PCVS, and underlying psychiatric conditions and to inform appropriate screening and treatment strategies.

## 5 Strengths and limitations

The main strength of this study lies in its large sample size, which enhances the generalizability of the findings to the general population. However, there are also methodological limitations that may affect the interpretability and validity of the results. Regarding the sample, it is important to note that only individuals aged 18 to 69 were surveyed. Adults over the age of 69—a high-risk group for severe COVID-19 outcomes—were not included in the study.

With respect to data collection, an obvious limitation is that the data reflects the views and self-assessments of the general population without any validation and classification of symptom severity by healthcare professionals. As such, personal opinions, beliefs, and attitudes toward COVID-19 and related public health measures may have influenced the results. Furthermore, detailed information on the temporal progression of symptoms was lacking. It was also not possible to accurately determine the temporal relationship between COVID infection and PCS symptoms or vaccine and PCVS symptoms. There was also no information on the persistence or recovery of symptoms over time and no information on the severity of symptoms. Understanding the course of these symptoms over time could be crucial in differentiating between PCS and PCVS. While long COVID is, by definition, a syndrome that persists for at least 3 months following infection, vaccine-related adverse effects typically occur acutely, shortly after vaccination. However, there are also delayed-onset vaccine side effects, which need to be distinguished from long COVID (32–34).

## Data Availability

The datasets presented in this study can be found in online repositories. The names of the repository/repositories and accession number(s) can be found in the article/Supplementary material.
