# Supplementary material for: Mental symptoms in post-COVID syndrome and post-COVID-19 vaccination syndrome: results of a representative population survey
Source: Front Public Health. 2025 Aug 4;13:1623757. doi: 10.3389/fpubh.2025.1623757 (PMC12358428; doi:10.3389/fpubh.2025.1623757)
Supplement: Supplementary file 1 [file Table_1.docx]

**S1 Table. Reported mental problems of PCS by gender and age (self-report, n=4,628, general population 18-69 years).**

|  |  | *n/total* | %w | [95% CI] | | OR | [95% CI] | | | *p* |
| --- | --- | --- | --- | --- | --- | --- | --- | --- | --- | --- |
|  | total | 560/4,628 | 12.1 | 11.1 | 13.2 |  |  | | | |
| gender | *female (=Ref.)* | *311/2,299* | *13.5* | *12.1* | *15.1* |  |  | | | |
|  | **male** | **249/2,329** | **10.7** | **9.4** | **12.1** | **0.77** | **0.64** | **0.91** | **0.0032** | |
| age | 18-29 y | 128/913 | 14.0 | 11.7 | 16.7 | 1.15 | 0.87 | 1.51 | 0.3217 | |
|  | *30-39 y (=Ref.)* | *113/908* | *12.4* | *10.3* | *15.0* | *1.00* |  | | | |
|  | 40-49 y | 95/835 | 11.4 | 9.2 | 13.9 | 0.90 | 0.68 | 1.21 | 0.4923 | |
|  | 50-59 y | 120/1,048 | 11.5 | 9.5 | 13.7 | 0.91 | 0.69 | 1.20 | 0.4984 | |
|  | **60-69 y** | **87/924** | **9.4** | **7.5** | **11.6** | **0.73** | **0.54** | **0.98** | **0.0382** | |

Percentages weighted by age, gender and federal state in Germany (%w, [95% confidence interval]) and odds ratios (OR). From N=4,628 people (general population 18-69 years). Significant differences are highlighted in bold. The reference categories are marked with (=Ref.).

**S2 Table. Reported mental symptoms of PCS (differentiated) by gender and age (self-report, n=4,628, general population 18-69 years).**

|  |  |  | *n/total* | %w | [95% CI] | | OR | [95% CI] | | | *p* |
| --- | --- | --- | --- | --- | --- | --- | --- | --- | --- | --- | --- |
| **tiredness, fatigue, weakness** |  | total | 434/4,628 | 9.4 | 8.5 | 10.3 |  | | | | |
|  | gender | *female (=Ref.)* | 247/2,299 | 10.7 | 9.5 | 12.2 |  | | | | |
|  |  | **male** | **187/2,399** | **7.8** | **6.7** | **9.0** | **0.73** | **0.59** | **0.89** | **0.0016** | |
|  | age | 18-29 y | 88/913 | 9.6 | 7.7 | 11.9 | 1.05 | 0.76 | 1.43 | 0.7774 | |
|  |  | *30-39 y (=Ref.)* | 84/908 | 9.3 | 7.4 | 11.5 |  |  | | | |
|  |  | 40-49 y | 87/835 | 10.4 | 8.4 | 12.9 | 1.14 | 0.83 | 1.56 | 0.8190 | |
|  |  | 50-59 y | 112/1,048 | 12.7 | 8.8 | 12.9 | 1.17 | 0.87 | 1.58 | 0.2919 | |
|  |  | 60-69 y | 63/924 | 6.8 | 5.2 | 8.7 | 0.72 | 0.51 | 1.01 | 0.0562 | |
| **restricted performance** |  | Total | 330/4,628 | 7.1 | 6.4 | 7.9 |  | | | | |
|  | gender | *female (=Ref.)* | 185/2,299 | 8.1 | 6.9 | 9.3 |  | | | | |
|  |  | **Male** | **145/2,329** | **6.2** | **5.3** | **7.3** | **0.76** | **0.61** | **0.95** | **0.0163** | |
|  | age | 18-29 y | 67/913 | 7.3 | 5.7 | 9.3 | 1.18 | 0.82 | 1.70 | 0.3693 | |
|  |  | *30-39 y (=Ref.)* | 57/908 | 6.3 | 4.8 | 8.1 |  | | | | |
|  |  | 40-49 y | 60/835 | 7.2 | 5.5 | 9.2 | 1.16 | 0.79 | 1.68 | 0.4495 | |
|  |  | 50-59 y | 86/1,048 | 8.2 | 6.6 | 10.1 | 1.33 | 0.94 | 1.89 | 0.1032 | |
|  |  | 60-69 y | 60/924 | 6.5 | 5.0 | 8.4 | 1.03 | 0.71 | 1.51 | 0.8501 | |
| **problems with concentration and memory** |  | Total | 292/4,628 | 6.3 | 5.6 | 7.1 |  | | | | |
|  | gender | *female (=Ref.)* | 172/2,299 | 7.5 | 6.4 | 8.7 |  | | | | |
|  |  | **Male** | **121/2,329** | **5.2** | **4.3** | **6.2** | **0.68** | **0.53** | **0.86** | **0.0015** | |
|  | age | 18-29 y | 70/913 | 7.7 | 6.0 | 9.7 | 1.24 | 0.86 | 1.78 | 0.2452 | |
|  |  | *30-39 y (=Ref.)* | 57/908 | 6.3 | 4.8 | 8.1 |  | | | | |
|  |  | 40-49 y | 47/835 | 5.6 | 4.1 | 7.5 | 0.89 | 0.60 | 1.33 | 0.5680 | |
|  |  | 50-59 y | 80/1,048 | 7.6 | 6.1 | 9.5 | 1.23 | 0.87 | 1.75 | 0.2419 | |
|  |  | 60-69 y | 40/924 | 4.3 | 3.1 | 5.9 | 0.68 | 0.45 | 1.02 | 0.0640 | |
| **sleep disorders** |  | Total | 231/4,628 | 5.0 | 4.4 | 5.7 |  | | | | |
|  | gender | *female (=Ref.)* | 139/2,299 | 6.0 | 5.1 | 7.1 |  | | | | |
|  |  | **Male** | **92/2,329** | **4.0** | **3.2** | **4.8** | **0.64** | **0.49** | **0.84** | **0.0012** | |
|  | age | 18-29 y | 42/913 | 4.6 | 3.3 | 6.2 | 1.16 | 0.74 | 1.84 | 0.5035 | |
|  |  | *30-39 y (=Ref.)* | 36/908 | 4.0 | 2.8 | 5.5 |  | | | | |
|  |  | 40-49 y | 43/835 | 5.1 | 3.7 | 6.9 | 1.32 | 0.84 | 2.07 | 0.2361 | |
|  |  | **50-59 y** | **65/1,048** | **6.2** | **4.8** | **7.9** | **1.60** | **1.06** | **2.43** | **0.0269** | |
|  |  | 60-69 y | 45/924 | 4.9 | 3.6 | 6.5 | 1.24 | 0.79 | 1.94 | 0.3467 | |
| **depressed mood, depression** |  | Total | 136/4,628 | 3.0 | 2.5 | 3.5 |  | | | | |
|  | gender | *female (=Ref.)* | 83/2,299 | 3.6 | 2.9 | 4.5 |  | | | | |
|  |  | **Male** | **53/2,329** | **2.3** | **1.7** | **3.0** | **0.62** | **0.44** | **0.88** | **0.0077** | |
|  | age | 18-29 y | 19/913 | 2.1 | 1.3 | 3.3 | 0.75 | 0.41 | 1.34 | 0.3518 | |
|  |  | *30-39 y (=Ref.)* | 25/908 | 2.8 | 1.8 | 4.1 |  | | | | |
|  |  | 40-49 y | 26/835 | 3.1 | 2.0 | 4.6 | 1.14 | 0.65 | 1.98 | 0.6557 | |
|  |  | 50-59 y | 40/1,048 | 3.8 | 2.7 | 5.2 | 1.40 | 0.84 | 2.33 | 0.1926 | |
|  |  | 60-69 y | 26/924 | 2.8 | 1.8 | 4.1 | 1.02 | 0.59 | 1.78 | 0.9372 | |

Percentages weighted by age, gender and federal state in Germany (%w, [95% confidence interval]) and odds ratios (OR). From N=4,628 people (general population 18-69 years). Significant differences are highlighted in bold. The reference categories are marked with (=Ref.).

**S3 Table. Reported mental side effects of COVID-19 vaccination (self-report, n=4.079 respondents with vaccination, population 18-69 years) by gender and age.**

|  |  | *n/total* | %w | [95% CI] | | OR | [95% CI] | | | *p* |
| --- | --- | --- | --- | --- | --- | --- | --- | --- | --- | --- |
|  | all persons with vaccination | 512/4,079 | 12.6 | 11.5 | 13.7 |  | | | | |
| gender | *female (=Ref.)* | *284/2,032* | *14.0* | *12.4* | *15.7* |  | | | | |
|  | male | 228/2,048 | 11.1 | 9.7 | 12.7 | 0.97 | 0.81 | 1.17 | 0.7627 | |
| age | 18-29 y | 117/802 | 14.6 | 12.1 | 17.4 | 1.28 | 0.96 | 1.72 | 0.0935 | |
|  | *30-39 y (=Ref.)* | *93/792* | *11.7* | *9.5* | *14.4* |  | | | | |
|  | 40-49 y | 96/711 | 13.5 | 10.9 | 16.5 | 1.17 | 0.86 | 1.59 | 0.3046 | |
|  | 50-59 y | 125/929 | 13.5 | 11.2 | 16.0 | 1.17 | 0.88 | 1.56 | 0.2873 | |
|  | 60-69 y | 81/845 | 9.6 | 7.6 | 11.9 | 0.80 | 0.58 | 1.09 | 0.1578 | |
| vaccine | *mRNA only* | 379/3,020 | 12.5 | 11.3 | 13.9 | 1.03 | 0.80 | 1.31 | 0.8331 | |
|  | *mRNA and non-mRNA (=Ref.)* | 91/742 | 12.3 | 9.9 | 15.1 |  | | | | |
|  | *Non-mRNA only* | 41/284 | 14.4 | 10.4 | 19.6 | 1.21 | 0.81 | 1.80 | 0.3529 | |
|  | *not specified/do not know* | 0/33 | 0.0 |  | | | | | | |
| number of vaccinations | ***1*** | **22/106** | **20.8** | **13.0** | **31.4** | **1.79** | **1.10** | **2.91** | **0.0183** | |
|  | *2* | 121/865 | 14.0 | 11.6 | 16.7 | 1.11 | 0.89 | 1.40 | 0.3555 | |
|  | *3 (=Ref.)* | 308/2,415 | 12.8 | 11.4 | 14.3 |  | | | | |
|  | ***4 or more*** | **62/693** | **8.9** | **6.9** | **11.5** | **0.67** | **0.50** | **0.90** | **0.0067** | |

Percentages weighted by age, gender and federal state in Germany (%w, [95% confidence interval]) and odds ratios (OR). From N=4,079 people (vaccinated against COVID-19, 18-69 years). Significant differences are highlighted in bold. The reference categories are marked with (=Ref.).

**S4 Table. Reported tiredness, fatigue, weakness as side effects of covid vaccination by gender, age, vaccine and number of vaccinations (self-report, n=4.079 respondents with vaccination, population 18-69 years).**

|  |  | *n/total* | %w | [95% CI] | | | OR | | [95% CI] | | *p* |
| --- | --- | --- | --- | --- | --- | --- | --- | --- | --- | --- | --- |
|  | all persons with vaccination | 366/4,079 | 9.0 | 8.1 | 10.0 |  | | | | | |
| gender | *female (=Ref.)* | 207/2,032 | 10.2 | 8.9 | 11.7 |  | | | | | |
|  | **Male** | **159/2,048** | **7.8** | **6.6** | **9.1** | **0.74** | **0.60** | **0.92** | | **0.0069** | |
| age | 18-29 y | 90/802 | 11.2 | 9.0 | 13.8 | 1.25 | 0.90 | 1.72 | | 0.1872 | |
|  | *30-39 y (=Ref.)* | 73/792 | 9.2 | 7.2 | 11.6 |  | | | | | |
|  | 40-49 y | 57/711 | 8.0 | 6.1 | 10.4 | 0.86 | 0.60 | 1.23 | | 0.4089 | |
|  | 50-59 y | 84/929 | 9.0 | 7.2 | 11.2 | 0.98 | 0.70 | 1.36 | | 0.8999 | |
|  | 60-69 y | 62/845 | 7.3 | 5.6 | 9.4 | 0.78 | 0.55 | 1.11 | | 0.1679 | |
| vaccine | *mRNA only* | 269/3,020 | 8.9 | 7.9 | 10.0 | 1.09 | 0.82 | 1.46 | | 0.5539 | |
|  | *mRNA and non-mRNA (=Ref.)* | 61/742 | 8.2 | 6.3 | 10.6 |  | | | | | |
|  | ***Non-mRNA only*** | **36/284** | **12.7** | **8.9** | **17.6** | **1.63** | **1.06** | **2.53** | | **0.0277** | |
|  | *not specified/do not know* | 0/33 | 0.0 |  | | | | | | | |
| number of vaccinations | ***1*** | **18/106** | **17.0** | **10.1** | **26.8** | **2.14** | **1.26** | **3.62** | | **0.0047** | |
|  | ***2*** | **96/865** | **11.1** | **9.0** | **13.6** | **1.30** | **1.01** | **1.68** | | **0.0412** | |
|  | *3 (=Ref.)* | 211/2,415 | 8.7 | 7.6 | 10.0 |  | | | | | |
|  | ***4 or more*** | **41/693** | **5.9** | **4.2** | **8.0** | **0.66** | **0.46** | **0.93** | | **0.0172** | |

Percentages weighted by age, gender and federal state in Germany (%w, [95% confidence interval]) and odds ratios (OR). From N=4,079 people (vaccinated against Covid, 18-69 years). Significant differences are highlighted in bold. The reference categories are marked with (=Ref.).

**S5 Table. Restricted performance as side effects of covid vaccination by gender, age, vaccine and number of vaccinations (self-report, n=4.079 respondents with vaccination, population 18-69 years).**

|  |  | *n/total* | %w | [95% CI] | | | OR | | [95% CI] | | *p* |
| --- | --- | --- | --- | --- | --- | --- | --- | --- | --- | --- | --- |
|  | all persons with vaccination | 176/4,079 | 4.3 | 3.7 | 5.0 |  | | | | | |
| gender | *female (=Ref.)* | 93/2,032 | 4.6 | 3.7 | 5.6 |  | | | | | |
|  | Male | 83/2,048 | 4.1 | 3.2 | 5.0 | 0.89 | 0.66 | 1.20 | | 0.4411 | |
| age | 18-29 y | 34/802 | 4.2 | 2.9 | 5.9 | 1.05 | 0.64 | 1.72 | | 0.8420 | |
|  | *30-39 y (=Ref.)* | 32/792 | 4.0 | 2.8 | 5.7 |  | | | | | |
|  | 40-49 y | 32/711 | 4.5 | 3.1 | 6.4 | 1.11 | 0.68 | 1.84 | | 0.6592 | |
|  | 50-59 y | 40/929 | 4.3 | 3.1 | 5.9 | 1.07 | 0.66 | 1.72 | | 0.7841 | |
|  | 60-69 y | 38/845 | 4.5 | 3.2 | 6.2 | 1.12 | 0.69 | 1.81 | | 0.6483 | |
| vaccine | *mRNA only* | 125/3,020 | 4.2 | 3.4 | 4.9 | 0.93 | 0.63 | 1.37 | | 0.7076 | |
|  | *mRNA and non-mRNA (=Ref.)* | 33/742 | 4.4 | 3.1 | 6.2 |  | | | | | |
|  | *Non-mRNA only* | 18/284 | 6.3 | 3.8 | 10.0 | 1.45 | 0.80 | 2.63 | | 0.2148 | |
|  | *not specified/do not know* | 0/33 | 0.0 |  | | | | | | | |
| number of vaccinations | **1** | **9/106** | **8.5** | **3.9** | **16.1** | **2.10** | **1.03** | **4.28** | | **0.0404** | |
|  | 2 | 46/865 | 5.3 | 3.9 | 7.1 | 1.27 | 0.89 | 1.82 | | 0.1843 | |
|  | 3 (=Ref.) | 102/2,415 | 4.2 | 3.4 | 5.1 |  | | | | | |
|  | 4 or more | 19/693 | 2.7 | 1.7 | 4.3 | 0.64 | 0.39 | 1.05 | | 0.0770 | |

Percentages weighted by age, gender and federal state (%w, [95% confidence interval]) and odds ratios (OR). From N=4,079 people (vaccinated against Covid, 18-69 years). Significant differences are highlighted in bold. The reference categories are marked with (=Ref.).

**S6 Table. Problems with concentration and memory as side effects of covid vaccination by gender, age, vaccine and number of vaccinations (self-report, n=4.079 respondents with vaccination, population 18-69 years).**

|  |  | *n/total* | %w | [95% CI] | | | OR | | [95% CI] | | *p* |
| --- | --- | --- | --- | --- | --- | --- | --- | --- | --- | --- | --- |
|  | all persons with vaccination | 149/4,079 | 3.7 | 3.1 | 4.3 |  | | | | | |
| gender | *female (=Ref.)* | 79/2,032 | 3.9 | 3.1 | 4.8 |  | | | | | |
|  | Male | 70/2,048 | 3.4 | 2.7 | 4.3 | 0.87 | 0.63 | 1.21 | | 0.4241 | |
| age | **18-29 y** | **28/802** | **3.5** | **2.3** | **5.0** | **0.60** | **0.37** | **0.97** | | **0.0383** | |
|  | *30-39 y (=Ref.)* | 45/792 | 5.7 | 4.1 | 7.6 |  | | | | | |
|  | 40-49 y | 30/711 | 4.2 | 2.8 | 6.0 | 0.73 | 0.46 | 1.17 | | 0.1952 | |
|  | **50-59 y** | **26/929** | **2.8** | **1.8** | **4.1** | **0.48** | **0.29** | **0.78** | | **0.0033** | |
|  | **60-69 y** | **20/845** | **2.4** | **1.4** | **3.7** | **0.40** | **0.24** | **0.69** | | **0.0009** | |
| vaccine | *mRNA only* | 99/3,020 | 3.3 | 2.7 | 4.0 | 1.01 | 0.64 | 1.60 | | 0.95212 | |
|  | *mRNA and non-mRNA (=Ref.)* | 24/742 | 3.2 | 2.1 | 4.8 |  | | | | | |
|  | ***Non-mRNA only*** | **26/284** | **9.2** | **6.0** | **13.4** | **3.01** | **1.70** | **5.35** | | **0.0002** | |
|  | *not specified/do not know* | 0/33 | 0.2 |  | | | | | | | |
| number of vaccinations | ***1*** | **10/106** | **9.4** | **4.5** | **17.3** | **3.12** | **1.56** | **6.22** | | **0.0012** | |
|  | ***2*** | **45/865** | **5.2** | **3.8** | **7.0** | **1.64** | **1.23** | **2.39** | | **0.0094** | |
|  | *3 (=Ref.)* | 78/2,415 | 3.2 | 2.6 | 4.0 |  | | | | | |
|  | ***4 or more*** | **16/693** | **2.3** | **1.3** | **3.7** | **0.71** | **0.41** | **1.22** | | **0.0214** | |

Percentages weighted by age, gender and federal state (%w, [95% confidence interval]) and odds ratios (OR). From N=4,079 people (vaccinated against Covid, 18-69 years). Significant differences are highlighted in bold. The reference categories are marked with (=Ref.).

**S7 Table. Sleep disorders as side effects of covid vaccination by gender, age, vaccine and number of vaccinations (self-report, n=4.079 respondents with vaccination, population 18-69 years).**

|  |  | *n/total* | %w | [95% CI] | | | OR | | [95% CI] | | *p* |
| --- | --- | --- | --- | --- | --- | --- | --- | --- | --- | --- | --- |
|  | all persons with vaccination | 139/4,079 | 3.4 | 2.9 | 4.1 |  | | | | | |
| gender | *female (=Ref.)* | 69/2,032 | 3.4 | 2.6 | 4.3 |  | | | | | |
|  | Male | 70/2,048 | 3.4 | 2.7 | 4.3 | 1.00 | 0.72 | 1.41 | | 0.9687 | |
| age | 18-29 y | 28/802 | 3.5 | 2.3 | 5.0 | 0.78 | 0.47 | 1.30 | | 0.3428 | |
|  | *30-39 y (=Ref.)* | 35/792 | 4.4 | 3.1 | 6.1 |  | | | | | |
|  | 40-49 y | 19/711 | 2.7 | 1.6 | 4.2 | 0.59 | 0.34 | 1.05 | | 0.0721 | |
|  | 50-59 y | 32/929 | 3,4 | 2.4 | 4.9 | 0.77 | 0.47 | 1.26 | | 0.2987 | |
|  | 60-69 y | 26/845 | 3.1 | 2.0 | 4.5 | 0.69 | 0.41 | 1.15 | | 0.1540 | |
| vaccine | *mRNA only* | 97/3,020 | 3.2 | 2.6 | 3.9 | 1.09 | 0.68 | 1.74 | | 0.7306 | |
|  | *mRNA and non-mRNA (=Ref.)* | 22/742 | 3.0 | 1.9 | 4.5 |  | | | | | |
|  | ***Non-mRNA only*** | **21/284** | **7.4** | **4.6** | **11.3** | **2.61** | **1.41** | **4.83** | | **0.0022** | |
|  | *not specified/do not know* | 0/33 | 0.0 |  | | | | | | | |
| number of vaccinations | ***1*** | **8/106** | **7.5** | **3.3** | **14.9** | **2.58** | **1.21** | **5.51** | | **0.0140** | |
|  | ***2*** | **40/865** | **4.6** | **3.3** | **6.3** | **1.53** | **1.04** | **2.27** | | **0.0328** | |
|  | *3 (=Ref.)* | 74/2,415 | 3.1 | 2.4 | 3.8 |  | | | | | |
|  | ***4 or more*** | 17/693 | 2.4 | 1.4 | 3.9 | 0.80 | 0.47 | 1.36 | | 0.4013 | |

Percentages weighted by age, gender and federal state (%w, [95% confidence interval]) and odds ratios (OR). From N=4,079 people (vaccinated against Covid, 18-69 years). Significant differences are highlighted in bold. The reference categories are marked with (=Ref.).

**S8 Table. Depressed mood/ depression as side effects of covid vaccination by gender, age, vaccine and number of vaccinations (self-report, n=4.079 respondents with vaccination, population 18-69 years).**

|  |  | *n/total* | %w | [95% CI] | | | OR | | [95% CI] | | *p* |
| --- | --- | --- | --- | --- | --- | --- | --- | --- | --- | --- | --- |
|  | all persons with vaccination | 63/4,079 | 1.5 | 1.2 | 2.0 |  | | | | | |
| gender | *female (=Ref.)* | 31/2,032 | 1.5 | 1.0 | 2.2 |  | | | | | |
|  | Male | 32/2,048 | 1.6 | 1.1 | 2.2 | 1.02 | 0.62 | 1.69 | | 0.9238 | |
| age | 18-29 y | 11/802 | 1.4 | 0.7 | 2.5 | 0.60 | 0.28 | 1.27 | | 0.1829 | |
|  | *30-39 y (=Ref.)* | 18/792 | 2.3 | 1.3 | 3.6 |  | | | | | |
|  | 40-49 y | 8/711 | 1.1 | 0.5 | 2.2 | 0.49 | 0.21 | 1.13 | | 0.0950 | |
|  | 50-59 y | 16/929 | 1.7 | 1.0 | 2.8 | 0.75 | 0.38 | 1.49 | | 0.4149 | |
|  | 60-69 y | 11/845 | 1.3 | 0.7 | 2.3 | 0.57 | 0.27 | 1.21 | | 0.1417 | |
| vaccine | *mRNA only* | 49/3,020 | 1.6 | 1.2 | 2.1 | 1.51 | 0.71 | 3.21 | | 0.2801 | |
|  | *mRNA and non-mRNA (=Ref.)* | 8/742 | 1.1 | 0.5 | 2.1 |  | | | | | |
|  | *Non-mRNA only* | 6/284 | 2.1 | 0.8 | 4.6 | 1.98 | 0.68 | 5.76 | | 0.2097 | |
|  | *not specified/do not know* | 0/33 | 0.0 |  | | | | | | | |
| number of vaccinations | **1** | **5/106** | **4.7** | **1.5** | **11.0** | **4.73** | **1.78** | **12.62** | | **0.0019** | |
|  | **2** | **26/865** | **3.0** | **2.0** | **4.4** | **2.96** | **1.70** | **5.16** | | **<0.0001** | |
|  | 3 (=Ref.) | 25/2,415 | 1.0 | 0.7 | 1.5 |  | | | | | |
|  | 4 or more | 7/693 | 1.0 | 0.4 | 2.0 | 0.98 | 0.42 | 2.27 | | 0.9540 | |

Percentages weighted by age, gender and federal state (%w, [95% confidence interval]) and odds ratios (OR). From N=4,079 people (vaccinated against Covid, 18-69 years). Significant differences are highlighted in bold. The reference categories are marked with (=Ref.).
